# An international survey of healthcare providers’ knowledge of cardiac complications of cancer treatments

**DOI:** 10.1186/s40959-019-0049-2

**Published:** 2019-09-02

**Authors:** J. Peng, M. Rushton, C. Johnson, C. Brezden-Masley, J. Sulpher, Miliyun G. Chiu, I. D. Graham, S. Dent

**Affiliations:** 1 Department of Medicine, University of Ottawa, Ottawa, ON Canada; 2 Department of Internal Medicine, University of Calgary, Calgary, AB Canada; 3 Division of Medical Oncology, Department of Medicine, The Ottawa Hospital Cancer Centre, Ottawa, ON Canada; 4 Division of Cardiology, Department of Medicine, The Ottawa Hospital, Ottawa, ON Canada; 5 Division of Medical Oncology, Department of Medicine, St. Michael’s Hospital, Toronto, ON Canada; 6 Division of Medical Oncology, Department of Medicine, BC Cancer Agency, Victoria, BC Canada; 7 Director of Peony Solutions, Kwai Bo Industrial Building, 40 Wong Chuk Hang Road, Aberdeen, Hong Kong; 8 School of Epidemiology and Public Health, University of Ottawa, Ottawa, ON Canada; 9 Division of Medical Oncology, Department of Medicine, Duke University, Durham, North Carolina USA

**Keywords:** Cardio-oncology, Knowledge translation, Cardio-toxicity, Guidelines, Cardiologists, Oncologists

## Abstract

**Background:**

Cardio-oncology is a young sub-specialty that addresses the needs of cancer patients at risk of, or who have experienced cancer therapy related cardiac dysfunction (CTRCD). This study assessed clinicians’ understanding of cardio-oncology, opinions towards current practice, and approach to diagnosing and managing CTRCD.

**Methods:**

A 45-question survey was administered online via Survey Monkey and WeChat to health care providers (HCPs) comprising of cardiologists, oncologists, and others from September 2017 to March 2018. Implementation of the survey followed a modified Dillman’s Total Design Method.

**Results:**

In total, 160 responses were collected from 22 countries; majority were from cardiologists (53.8%) and oncologists (32.5%). The remaining 13.7% identified themselves as “others,” including general internists, cardio-oncologists, pediatric oncologists, radiation oncologists, cardiac rehabilitation therapists, nurse practitioners, research students, and pharmacists. In the setting of metastatic cancer, there was a difference in risk tolerance for cardiotoxicity between subspecialties. In this case, more cardiologists (36.7%) accepted a 5–10% risk of cardiotoxicity compared to oncologists (20.0%). Majority of cardiologists felt that cardiotoxicity should be monitored, even in asymptomatic cancer patients (55.8%). Only 12% of oncologists selected this response. In contrast, 50.0% of oncologists reported that cardiologists should be involved only when patients develop cardiotoxicity. In comparison, 6.5% of cardiologists selected this response. Majority of cardiologists stated that cardio-oncology clinics would significantly improve cancer patients’ prognosis (88.3%); only 45.8% of oncologists shared this opinion. Of all respondents, 66.9% stated they were familiar with a variety of international guidelines for managing cardiotoxicity. Of all oncologists, 65.3% indicated that they referred to these guidelines for clinical decision making.

**Conclusions:**

Despite the growth of cardio-oncology clinics, there are significant knowledge gaps regarding prevention and treatment strategies for CTRCD among health care providers. Knowledge translation from guidelines and collaboration between cardiologists and oncologists are needed to improve cardiovascular outcomes of cancer patients.

**Electronic supplementary material:**

The online version of this article (10.1186/s40959-019-0049-2) contains supplementary material, which is available to authorized users.

## Introduction

Cancer and cardiovascular disease are the leading causes of morbidity and mortality in Canada and the United States [1, 2]. With early detection and improvements in cancer treatment, an increasing number of individuals are surviving a cancer diagnosis [3]. By 2020 it is estimated there will be over 18 million cancer survivors in the United States alone [4]. Cancer treatments, however, can have a deleterious impact on the cardiovascular system, including: myocardial dysfunction, systemic hypertension, QT prolongation, arrhythmias, thromboembolic events, pericardial and valvular heart disease [5]. In the years after curative breast cancer treatment, post-menopausal women have a greater risk of dying of cardiovascular disease than recurrence of their cancer—in part due to baseline risk factors that may be potentiated by cancer treatment related cardiac dysfunction (CTRCD) [6].

Cardio-oncology is an emerging speciality focused on the cardiovascular care of cancer patients and cancer survivors. The scope of cardio-oncology includes pre-cancer treatment optimization, diagnosis, and management of cardiac complications of cancer treatment during and following completion of cancer treatment. A number of position statements and guidelines in cardio-oncology have been published by international organizations including: the American Society of Clinical Oncology (ASCO), European Society of Cardiology (ESC), and the Canadian Cardiovascular Society (CCS) to provide guidance on the detection and management of CTRCD [7–9]. While these efforts should be applauded, there is no data examining the uptake of these guidelines in clinical practice. Education of health care providers (HCPs), specifically, cardiologists, oncologists, general internists, primary care providers, or nurse practitioners, has been facilitated by organizations such as the Canadian Cardiac Oncology Network (CCON) and the International Cardio-Oncology Society (ICOS) through conferences and continuing medical education events; however the impact of these educational initiatives is not clear [10, 11]. The lack of formal training programs in cardio-oncology has limited the knowledge uptake in this field [12].

The objective of this international cardio-oncology survey was to gain a better understanding of the current knowledge of HCPs tasked with caring for cancer patients with CTRCD. We specifically targeted cardiologists and oncologists from sites within and outside of North America. Information from this study will inform cardio-oncologists and cardio-oncology training programs of existing knowledge gaps and help to direct future educational and research efforts.

## Methods

### Study design and sample

Between September 2017 to March 2018, we conducted a 45-question online survey of HCPs globally. For this study, HCPs included cardiologists, oncologists, and others such as general internists, cardio-oncologists, pediatric oncologists, radiation oncologists, cardiac rehabilitation therapists, nurse practitioners, research students, and pharmacists. The survey was conducted via Survey Monkey in North and South America, Europe, and India, and via WeChat in China; in total, 22 countries were included (Additional file 1: Appendix 1). A cover letter described the research study and included a SurveyMonkey or Wechat link that provided access to the survey. The letter was openly distributed by major cardiology and oncology societies – specifically, at the 2017 Global Cardio-Oncology Symposium (GCOS), the 2017 Canadian Cardiology Conference (CCC), in the CCS Member Bulletin, and by the Canadian Association of Medical Oncologists (CAMO). Follow up reminders were sent at 1, 3, and 7 weeks. A modified Dillman’s total design method was used to maximize responses [13]. By completing the survey, implied consent was provided to use responses for the research study. This study was approved by the Ottawa Health Science Network Research Ethics Board at the Ottawa Hospital Research Institute.

### Survey format

The survey questions were initially drafted by two oncologists and two cardiologists specializing in cardio-oncology. The survey consisted of 45 questions that were organized into 7 sections, including multiple choice questions, 5-point Likert scale questions, and two clinical scenarios (outlined in the Additional file 1: Appendix 2). The survey questions were pilot tested among cardiologists and oncologists in Ottawa. Of the 45 questions, 5 were directed specifically to cardiologists and 13 were directed specifically to oncologists. Section I focused on the demographics of respondents. Section II assessed the respondent’s perception of cardio-oncology, such as asking about one’s definition of “cardio-oncology.” Section III evaluated the availability of cardio-oncology services at the respondent’s institution. Section IV asked about opinions towards current practice, evaluating whether it was important for oncologists to consider cardiotoxicity when planning, using, or completing cancer therapy; gauging when cardiologists should be involved in a cancer patient’s care; and assessing whether cardio-oncology clinics have an impact on cancer patients’ prognosis. Section V solely targeted cardiologists to evaluate their knowledge and comfort with identifying and treating cardiotoxicity, and determine whether their oncology colleagues were skilled in this aspect. Section VI solely targeted oncologists to evaluate their knowledge and comfort with identifying and treating cardiotoxicity, and determine whether their cardiology colleagues were skilled in this aspect. Section VII included two clinical scenarios that addressed common cardiotoxicity issues in oncology.

### Data analysis

Responses from China that were collected via WeChat were entered manually into the Survey Monkey database. Thus, responses from China were analyzed together with those from other countries. For analysis, Survey Monkey was used to present descriptive quantitative data. Filters for “Cardiologist” and “Oncologist” were created for questions of interest to determine whether there was a difference in opinion between the two specialties. Response rate was not calculated given the survey was openly distributed via society newsletters and conferences rather than individually emailed.

## Results

### Demographics of respondents and experience in practice

A total of 160 responses were collected from 22 countries, including Canada (*n* = 50), China (*n* = 35), USA (*n* = 20), and Brazil (*n* = 11). The majority of respondents were cardiologists (53.8%) followed by oncologists (32.5%) (see Table 1); the remaining 13.7% of respondents included “others” who were general internists (3.1%), cardio-oncologists, pediatric oncologists, radiation oncologists, cardiac rehabilitation therapists, nurse practitioners, research students, and pharmacists. Most respondents were attending physicians (75.0%), with 41.8% having been in practice for over 20 years since completing residency. The majority (76.3%) of participants worked at an academic institution. Of all respondents, only 16.9% (*n* = 27) reported having some training in cardio-oncology, primarily in the form of conferences (*n* = 11/27) or a fellowship training program (*n* = 7/27).

**Table 1:** Demographics of health care professionals surveyed

Health Care Professional *N* = 160% of responses		
Cardiologist	86	53.8%
Medical Oncologist	52	32.5%
Other	22	13.7%
Practice Setting		
Tertiary care hospital	122	76.3%
Secondary hospital	24	15.0%
Private office	7	4.4%
Other	7	4.4%

### Perception of cardio-oncology and cardiotoxicity

**Fig. 1:**
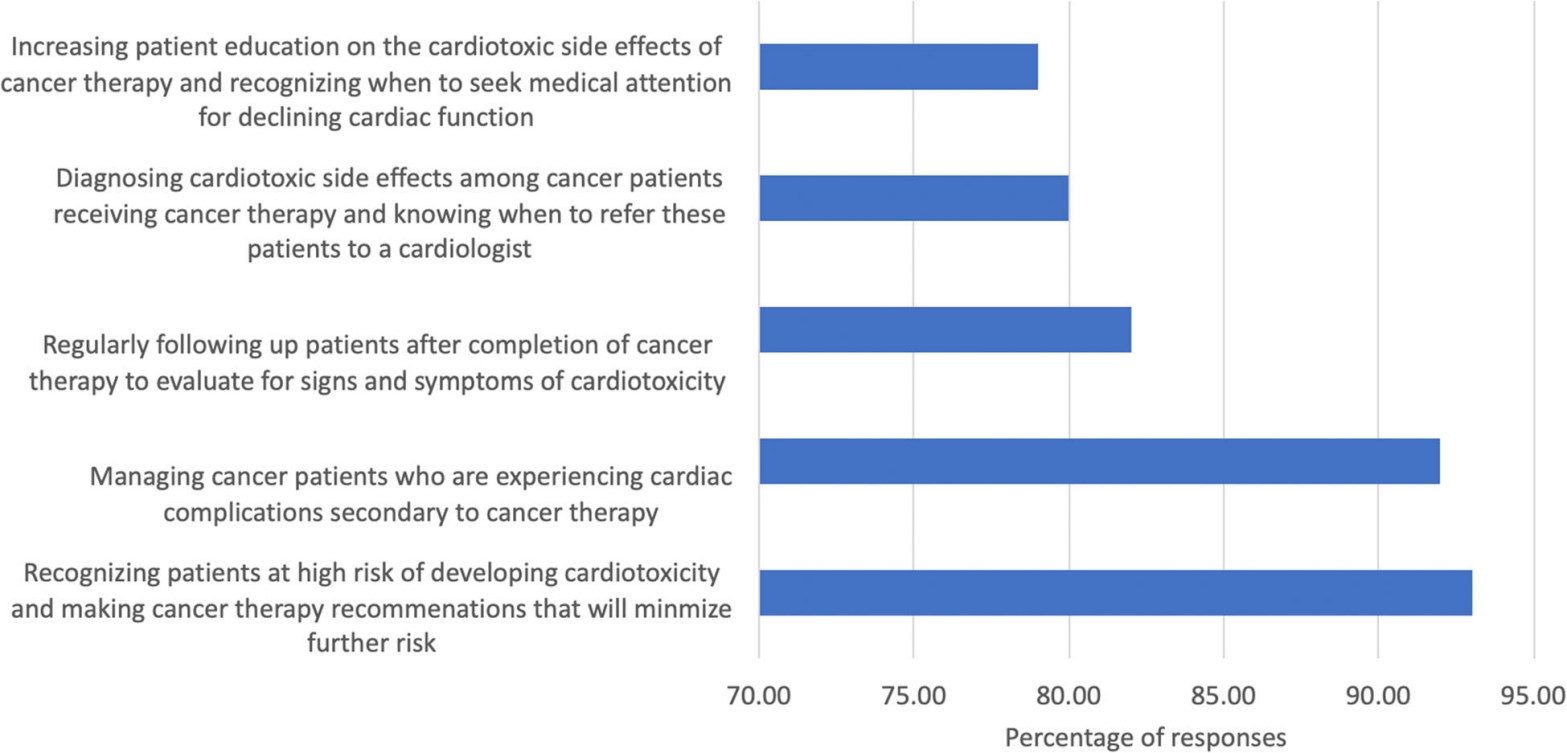
Perception of of cardio-oncology: Survey responses on the perception of cardio-oncology by heart health practitioners. Note that respondents were able to select as many answers as they felt appropriate

Respondents were asked in a multiple-choice question what “cardio-oncology” meant to them as a discipline. Recognizing patients at high risk of developing cardiotoxicity and choosing a cancer therapy that will minimize this risk was a prominent response (92.7%). Additionally, 92.0% of respondents believed that cardio-oncology pertained to the management of patients experiencing CTRCD. Fewer respondents selected diagnosing cardiotoxicity (80.0%) and following patients for signs and symptoms of cardiotoxicity (82.0%) as the emphasis of cardio-oncology. (see Fig. 1).

**Fig. 2:**
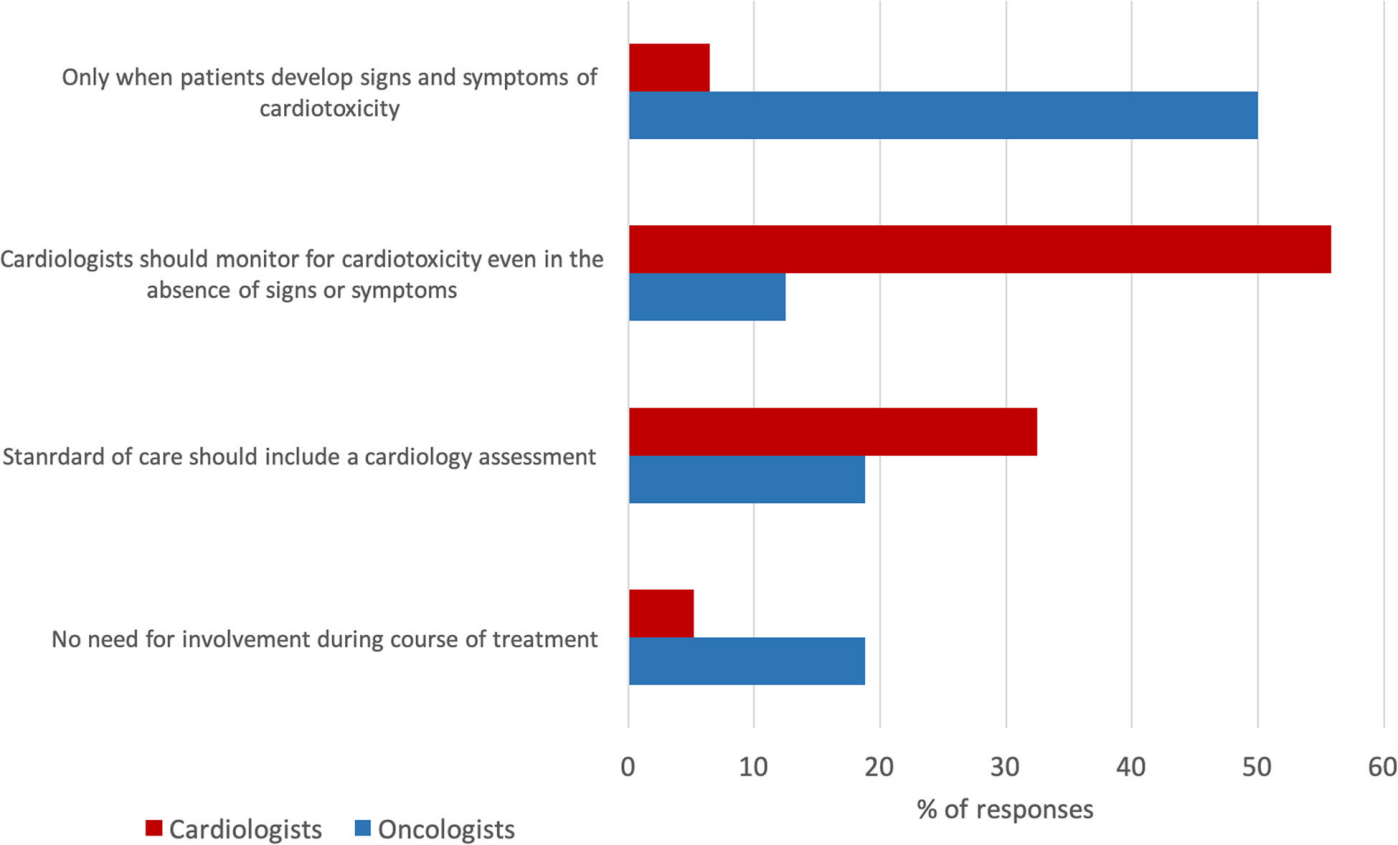
Involvement of a cardiologist in a cancer patient’s care: For a patient with no underlying cardiac issues who is being started on cancer therapy with potential cardiotoxic side effects, when should a cardiologist be involved?

Cardiologists and oncologists held different opinions regarding the value of cardio-oncology clinics or when cardiologists should be involved in caring for cancer patients. The majority of cardiologists felt that they should regularly monitor for cardiotoxicity in cancer patients, even in the absence of symptoms (55.8%). Only 12.5% of oncologists selected this answer. In contrast, 50.0% of oncologists felt that cardiologists should be involved only when patients developed cardiotoxicity. Only 6.5% of cardiologists selected this answer. (see Fig. 2). The majority of cardiologists believed that access to cardio-oncology services would significantly improve cancer patients’ prognosis (88.3%). Comparatively, 45.8% of oncologists shared this opinion.

### Evaluation of current practice and availability of training programs

**Fig. 3:**
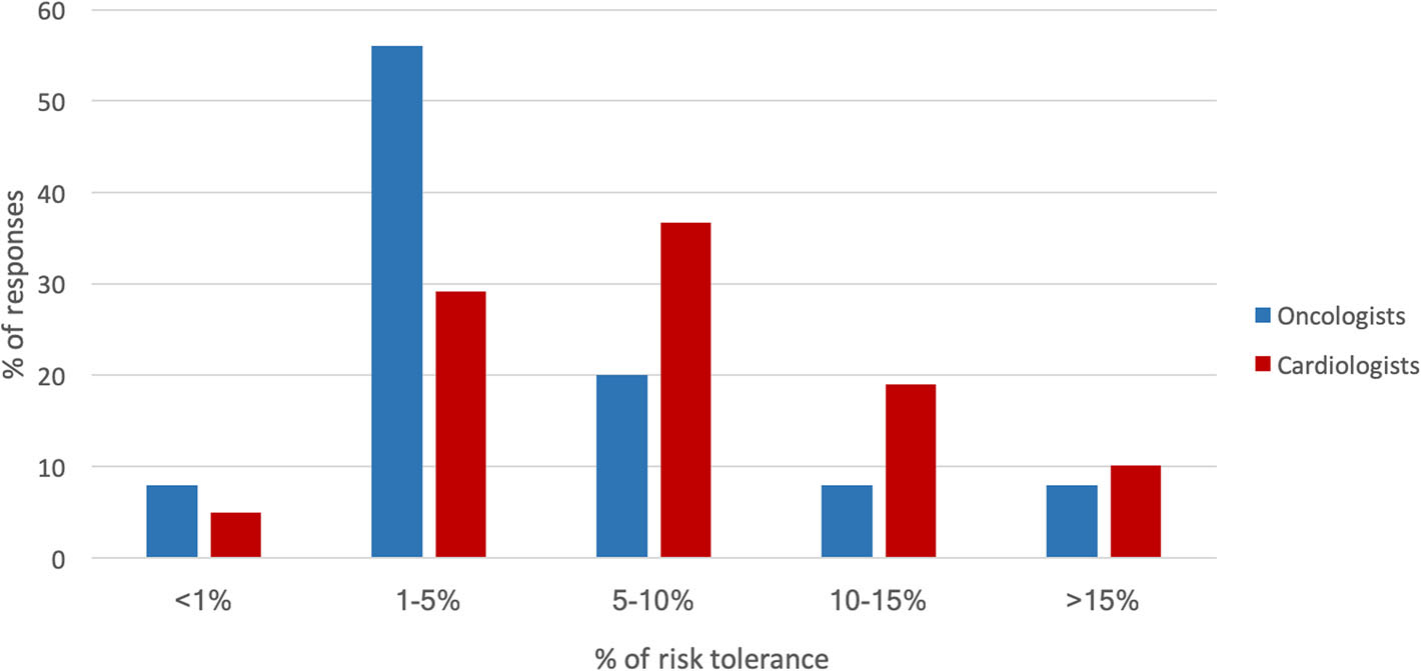
Acceptable risk of cardiotoxicity: Risk tolerance of cardiotoxicity in the metastatic setting

Several cardiologists strongly agreed they were knowledgeable about cardiotoxic complications from cancer therapy (48.8%; *n* = 42) and were comfortable treating these complications (41.0%; *n* = 35). In contrast, only 8.2% of oncologists strongly agreed that cardiologists were knowledgeable about cardiotoxicity and were comfortable treating these complications. Very few (2.1%) oncologists strongly agreed that they were comfortable treating cardiovascular complications of cancer therapy. There was also a difference in risk tolerance for cardiotoxicity between specialties. In the setting of metastatic cancer, more cardiologists (36.7%) accepted a higher risk of cardiotoxicity (5–10% risk) compared to oncologists (20.0%) (see Fig. 3).

Regarding education in cardio-oncology, 38.0% of all respondents reported that formal training in cardio-oncology was lacking. Respondents believed that barriers to the development of cardio-oncology clinics were due to limited funding (68.0%) and limited infrastructure (54.0%).

Of all respondents, 66.9% reported that they were familiar with guidelines from expert societies for managing cardiotoxicity. Among those who were familiar with guidelines, many cited resources from ESC (43.6%). When asked about utilizing resources, 65.3% of oncologists stated that they referred to these international guidelines for clinical decision making.

### Responses to clinical scenarios

**Fig. 4:**
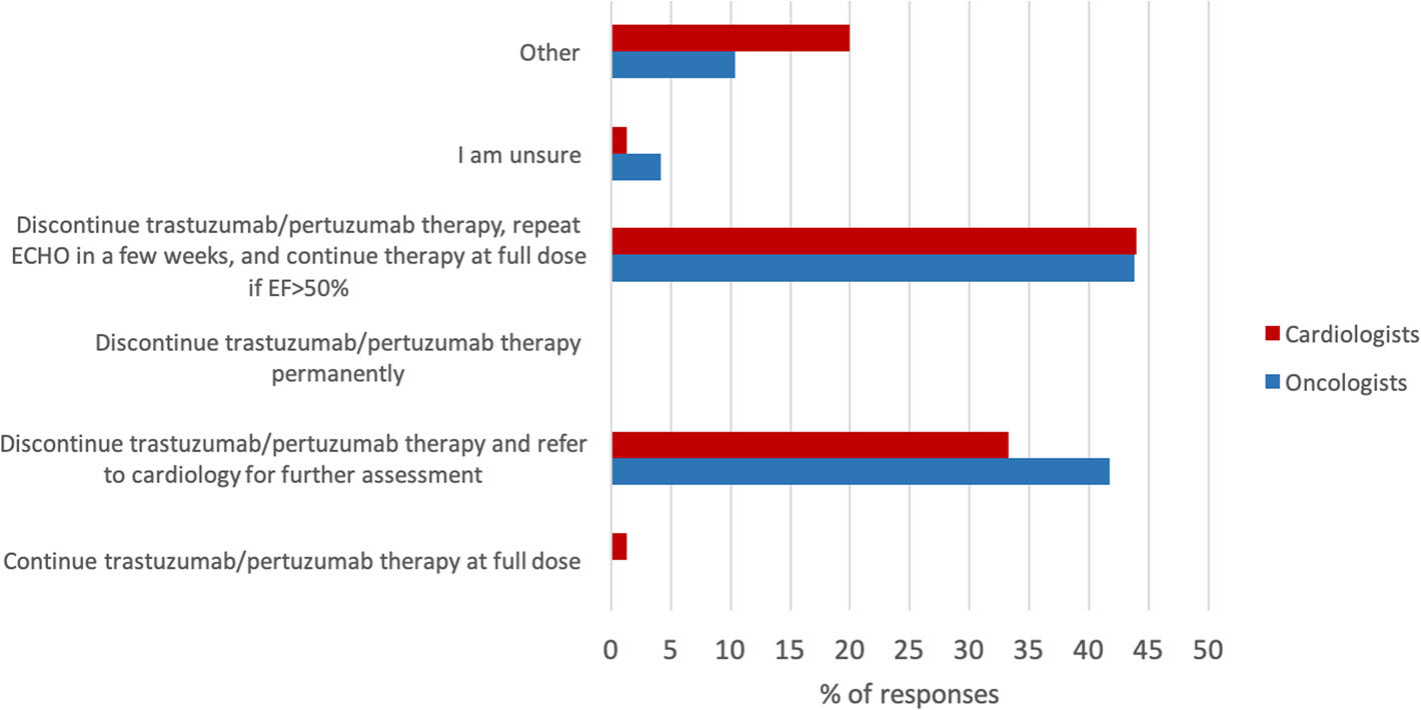
Responses to clinical scenario #1**:** A 50 year old female has received 12 cycles of trastuzumab/pertuzumab therapy for Her-2/neu positive metastatic breast cancer. Her ejection fraction at baseline was 55%, but on repeat echocardiogram decreased to 30%. She has no cardiac symptoms. What would be your management of the patient at this time?

**Fig. 5:**
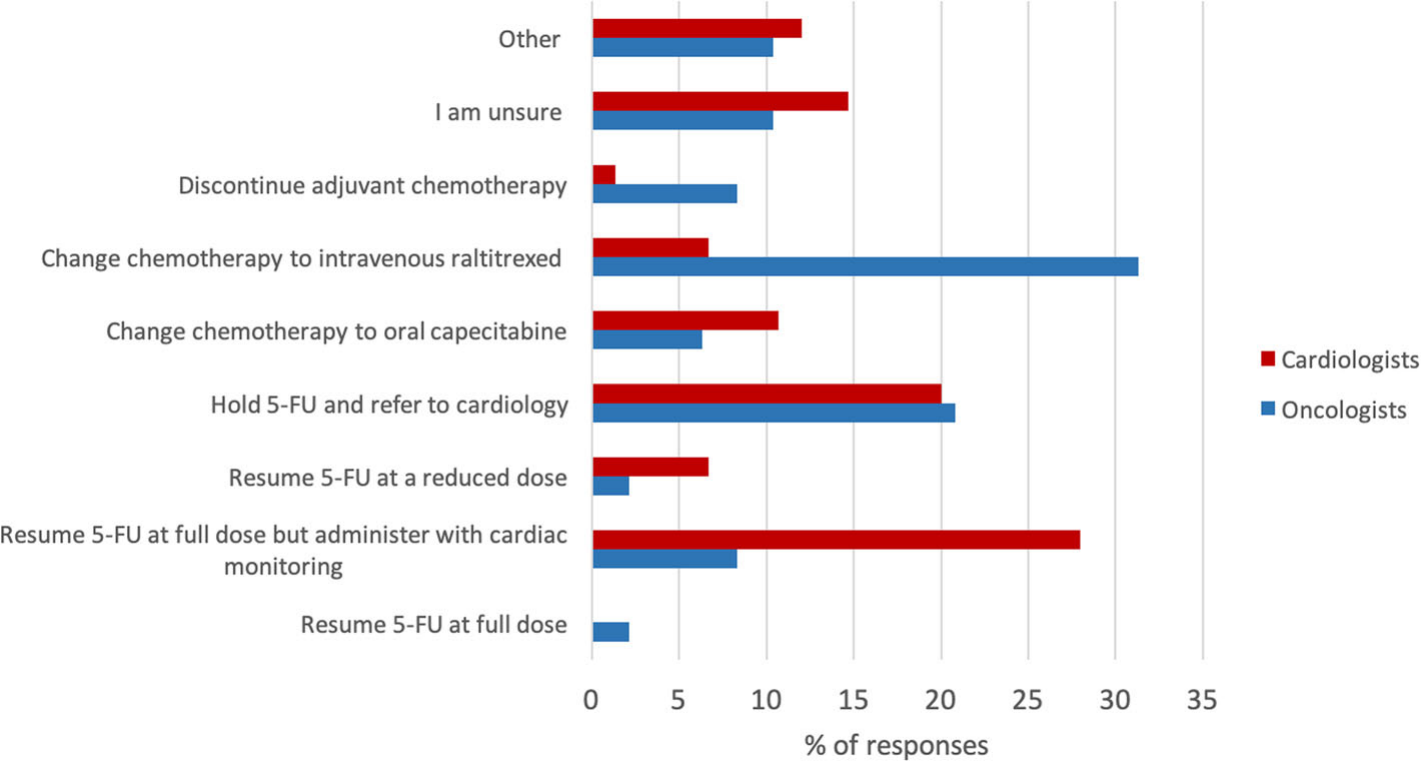
Responses to clinical scenario #2: A 58 year old male is receiving adjuvant infusional 5-fluorouracil during cycle 2 for resected stage III colorectal carcinoma. He develops sudden chest pain and nausea, and presents to the emergency department. A 12 lead electrocardiogram reveals inferior ST segment elevation. He is managed medically with complete resolution of symptoms. A subsequent angiogram reveals no evidence of coronary artery disease. A follow-up echocardiogram reveals an ejection fraction of 58%. What would you now recommend for adjuvant chemotherapy?

The first clinical scenario described a patient with human epidermal growth factor receptor 2 positive (HER2+) breast cancer who experienced an asymptomatic decline in left ventricular ejection fraction (LVEF) (55 to 33%) while receiving trastuzumab. Guidelines for holding trastuzumab in the presence of an asympotmatic drop in LVEF are based on adjuvant trastuzumab clinical trials[14].  The National Cancer Research Institute recommends interruption of trastuzumab if the LVEF decreases to < 45% and reinitiation of treatment when the LVEF recovers to > 49% [15]. This evidence-based answer was selected by 44.0% of cardiologists and 43.8% of oncologists (see Fig. 4). The second clinical scenario described a patient with resected colorectal cancer who developed chest pain and ECG changes (ST segment elevation in inferior leads) while on infusional 5-fluorouracil (5-FU) chemotherapy. As per CCS guidelines, if there is a temporal relationship between 5-FU and angina, 5-FU should be stopped. If there is myocardial ischemia associated with 5-FU, another cancer therapy should be considered and re-challenge with 5-FU is generally not recommended due to the high risk of recurrent ischemia [9]. There was less consistency in responses in this scenario. Approximately 28% of cardiologists chose to resume 5-FU at full dose with cardiac monitoring. Among oncologists, 31.3% elected to change the chemotherapy to intravenous raltitrexed while 20.8% of oncologists elected to hold 5-FU and refer to cardiology. Only 6.7% of cardiologists chose to change adjuvant chemotherapy to raltitrexed, another therapeutic option (see Fig. 5).

## Discussion

Despite the emergence of cardio-oncology programs globally, the consequences of CTRCD remain incompletely understood by many cardiologists, oncologists, and primary care providers in the community. Previous studies have explored the knowledge of HCPs in cardio-oncology; however, they were limited in scope [16–18]. Barac et al. conducted a nationwide survey in the United States limited to cardiologists, while Jovenaux et al. conducted a cross-sectional survey of oncologists from across France [17, 18]. Our group previously conducted a survey of cardiologists and oncologists in 2015, however, the majority of participants were from North America [16].

For this study, we conducted an international survey to determine the current perception, practices, and knowledge of CTRCD among HCPs globally. While the majority of cancer care is delivered in the community, respondents were largely from academic institutions [19]. This likely reflects the novelty of cardio-oncology as a sub-speciality, as well as the limited number of HCPs with expertise in this area. Future directions should include the education and training of HCPs in the community, as well as improvement in patient access to cardio-oncology services through use of modern technologies such as tele-medicine and tele-health [19].

The clinical value of cardio-oncology clinics was perceived differently between HCPs. Cardiologists felt strongly that access to cardio-oncology clinics would improve prognosis for cancer patients; oncologists were less convinced. These findings are similar to a previous survey of U.S. based cardiologists [18]. While there has been growth of cardio-oncology clinics globally, information on whether cardio-oncology clinics ‘really’ improve care is lacking. Research to define qualitative (e.g patient satisfaction) and quantitative measures (e.g completion of cancer therapy; prevention of heart failure) for cardio-oncology clinics is needed to determine the short and long term benefits) of this multidisciplinary approach.

Cardiologists were more likely than oncologists to recommend early referral of cancer patients to a cardio-oncology clinic. Studies have shown that patients with one or more cardiovascular risk factors are more likely to experience a cardiac event when exposed to cancer therapy [20, 21]. Anthracyclines, a cornerstone of cancer treatment, are associated with increased risk of irreversible cardiotoxicity, and ultimately, cardiomyopathy [22]. However, Cardinale et al. demonstrated that if anthracycline induced cardiotoxicity is detected early (< 6 months from insult), medical intervention can reverse cardiac damage; thereby, supporting early detection and management of high risk patients [23]. Early referral to a cardio-oncology clinic may also improve implementation of primary prevention strategies to reduce the risk of cardiotoxicity. Primary prevention strategies for individuals at highest risk of CTRCD are currently being explored. In breast cancer patients, there is emerging evidence for prescribing beta-blockers or angiotensin converting enzyme (ACE) inhibitors upfront in patients receiving chemotherapy and/or trastuzumab for prevention of CTRCD [24–26].

Our study indicated that compared to oncologists, cardiologists were more accepting of a higher risk of cardiotoxicity, especially in the setting of advanced disease. This likely reflects the expertise of the cardiologists who completed this survey. Oncologists, even in the academic setting, may feel less comfortable with this approach, perhaps due to a less comprehensive understanding of potential treatment options available to mitigate cardiotoxicity in these patients. In addition, CTRCD remains a largely unfamiliar topic among many oncologists who practice in the community. This places cancer patients at risk of permanent discontinuation of life saving or sustaining cancer therapy. Education of oncologists, cardiologists, and allied health care providers on the impact of CTRCD and strategies to mitigate and treat CTRCD should be supported by institutions.

There is limited information on the utilization of cardio-oncology guidelines in clinical practice—an area our survey attempted to address. Responses to our two clinical cases suggest that HCPs were selecting evidence-based answers more frequently than in previous studies [16]. In the first case, almost half of respondents selected the evidence-based answer to discontinue trastuzumab in a patient with an LVEF < 50%, repeat an echocardiogram (ECHO), and continue therapy at full dose if the LVEF normalized [15, 27]. This is an improvement from a previous survey, where only 21% of oncologists resumed trastuzumab in an asymptomatic patient with a LVEF of 40–50% [16]. Familiarity with the literature highlighting the reversibility of trastuzumab-associated cardiotoxicity; use of biomarkers to predict cardiotoxicity in patients receiving cancer therapy; and increased comfort among cardiologists in managing cardiotoxicity, likely account for these changing results in our survey [28–30]. The second case described the most common manifestation of 5-FU cardiotoxicity: angina [31–33]. More oncologists decided to switch therapy to raltitrexed rather than re-challenge with 5-FU. In this case, oncologists’ preference to switch to raltitrexed is supported by current guidelines, although centers with expertise in cardio-oncology are now re-challenging patients with 5-FU using strict protocols [9, 34, 35].

This study was not without limitations. We had a lower than expected number of responses, receiving only 160 responses despite reaching out to major cardiology and oncology associations. For future studies, personalized contact rather than open distribution of the survey link may improve response rates. We were also not able to assess regional differences due to limited responses per country. There was a component of sampling bias, where opinions reflected in this survey were primarily those of attending physicians working at academic centres. The opinions of physicians who practice in the community may suggest different values (e.g. risk tolerance for cardiotoxicity, when specialists should be involved in a cancer patient’s care) and uptake of guidelines in cardio-oncology. It is conceivable that the survey had higher uptake by specialists who were interested and experienced in the field of cardio-oncology; HCP’s were less likely to participate if they did not have confidence in their knowledge of this field. These limitations should inform clinicians on the importance of ongoing educational campaigns and updated guidelines to assist in clinical decision making.

## Conclusion

Cancer and Cardiovascular disease are the leading causes of morbidity and mortality in developed countries. The complexity of caring for cancer patients who develop CTRCD or patients with pre-existing cardiovascular disease who develop cancer will continue to increase. Cardio-oncology has emerged as a new field to address the needs of these patients; however, it remains an unknown entity for many HCPs and patients. There is a need to educate HCPs and cancer patients on the impact of cancer therapy on cardiovascular health. Our survey results, although limited due to the small number of respondents, still identified knowledge gaps and differing opinions in this field. Several societies, including the International Cardio-Oncology Society (ICOS), the Canadian Cardiac Oncology Network (CCON) and the British Cardio-Oncology Society (BCOS) have emerged to foster the clinical care, education, and research in this field. Future studies should address knowledge gaps among community HCPs who are tasked with providing the majority of cancer care in North America and globally.

## Additional file

Additional file 1: Appendix 1: List of respondents per country. Appendix 2: Cardio-Oncology Survey. (DOCX 87 kb)

## Data Availability

The datasets used and/or analyzed during the current study are available from Survey Monkey upon reasonable request to the corresponding author.

## Abbreviations

5-FU: 5-fluorouracil
ACE: Angiotensin converting enzyme
ASCO: American Society of Clinical Oncology
BCOS: British Cardio-Oncology Society
CAMO: Canadian Association of Medical Oncologists
CCC: Canadian Cardiology Conference
CCON: Canadian Cardiac Oncology Network
CCS: Canadian Cardiovascular Society
CTRCD: Cancer therapy related cardiac dysfunction
ECG: Electrocardiogram
ECHO: Echocardiogram
ESC: European Society of Cardiology
GCOS: Global Cardio-Oncology Symposium
HCPs: Health care providers
HER2 +: Human epidermal growth factor receptor 2positive
ICOS: International Cardio-oncology Society
LVEF: Left ventricular ejection fraction
