# Supplementary material for: An international survey of healthcare providers’ knowledge of cardiac complications of cancer treatments
Source: Cardiooncology. 2019 Sep 2;5:12. doi: 10.1186/s40959-019-0049-2 (PMC7048147; doi:10.1186/s40959-019-0049-2)
Supplement: Supplementary file 1 — Appendix 1: List of respondents per country. Appendix 2: Cardio-Oncology Survey. (DOCX 87 kb) [file 40959_2019_49_MOESM1_ESM.docx]

**APPENDICES:**

**Additional file 1: Appendix 1: List of respondents per country:**

- Canada: 50
- China: 35
- USA: 20
- United Kingdom: 16
- Brazil: 11
- Colombia: 3
- Spain: 3
- Australia: 2
- Denmark: 2
- Dominican Republic: 2
- Estonia: 2
- Ireland: 2
- Italy: 2
- Turkey: 2
- Argentina: 1
- Ecuador: 1
- Greece: 1
- India: 1
- Peru: 1
- Poland: 1
- Saudi Arabia: 1
- South Africa: 1

**Additional file 1: Appendix 2: Cardio-Oncology Survey**

**SECTION I – DEMOGRAPHICS**

1. Please state the country in which you practice (drop down menu)
2. Please state your specialty (drop menu format)
   1. Cardiology
   2. Oncology
   3. Internal Medicine
   4. Other (please specify)
3. Does your practice location have the following (select as many as apply)
   1. Cardiology training program
   2. Medical Oncology training program
   3. Cardio-oncology training program
   4. None of the above
4. How would you best describe your practice location?
   1. Tertiary care hospital (access to full range of medical and surgical specialists)
   2. Secondary hospital (hospital with limited specialized care)
   3. Private office
   4. Other (please explain)
5. What percentage of your time is spent on clinical research? (Fill in the blank)
6. What is your current position?
   1. Attending physician
   2. Clinical fellow
   3. Resident physician (please state year of residency)
7. If you answered “attending physician” in question 6, how many years have you been in practice since completing residency?
   1. 0 – 5 years
   2. 6 – 10 years
   3. 11 – 20 years
   4. >20 years
8. Have you previously received any formal training in cardio-oncology?
   1. Yes
   2. No
9. If you answered “yes” in question 8, please specify the type of training received.

**SECTION II – PERCEPTIONS OF CARIO-ONCOLOGY**

1. What does the field of “Cardio-Oncology” mean to you? Please select all that apply.
   1. D*iagnosing* cardiotoxic side effects among cancer patients receiving cancer therapy and knowing when to refer these patients to a cardiologist
   2. *Managing* cancer patients who are experiencing cardiac complications secondary to cancer therapy
   3. Recognizing patients at high risk of developing cardiotoxicity and making *cancer therapy recommendations* that will minimize further risk
   4. Regularly following up with patients after completion of cancer therapy to evaluate for signs and symptoms of cardiotoxicity
   5. Increasing patient education on the cardiotoxic side effects of cancer therapy and recognizing when to seek medical attention for declining cardiac function
2. Based on your clinical expertise, when are patients at the greatest risk of experiencing cardiotoxicity from targeted therapies (e.g. trastuzumab)?
   1. During cancer treatment (chemotherapy and/or targeted agents)
   2. 1 year post cancer therapy (short term risk)
   3. 1 to 5 years post cancer therapy
   4. Greater than 5 years post cancer therapy (long term risk)
3. Based on your clinical expertise, when are patients at greatest risk of experiencing cardiotoxicity from chemotherapy (e.g. anthracyclines)?
   1. During cancer treatment (chemotherapy and/or targeted agents)
   2. 1 year post cancer therapy (short term risk)
   3. 1 to 5 years post cancer therapy
   4. Greater than 5 years post cancer therapy (long term risk)
4. What do you consider to be an acceptable risk of cardio-toxicity for *your patients* when consenting for cancer therapy in the curative setting?
   1. <1%
   2. 1-5%
   3. 5-10%
   4. 10-15%
   5. >15%
5. What do you consider to be an acceptable risk of cardio-toxicity for *your patients* when consenting for cancer therapy in the metastatic (incurable) setting?
   1. <1%
   2. 1-5%
   3. 5-10%
   4. 10-15%
   5. >15%
6. Are you familiar with guidelines from expert societies on the direction and management of cardiovascular toxicities?
   1. Yes
   2. No
7. If you answered “yes” to question 15, please specify which organization/society

**SECTION III – AVAILABILITY OF CARDIO-ONCOLOGY SERVICES AT YOUR INSTITUTION**

1. At your current institution, what *education programs* are available in the field of cardio-oncology? Please select all that apply.
   1. There is little cardio-oncology training for clinical cardiology fellows
   2. Cardiology fellows are exposed to cardio-oncology during clinical rotations as part of the curriculum
   3. There are some lectures on cardio-oncology as part of the core curriculum or CME offerings
   4. Cardiology fellows can *choose* to spend time training with the oncology service but this is not part of the core curriculum (please specify duration of training)
   5. There is a dedicated cardio-oncology fellowship program (at least 6 months)
   6. I am unsure of available cardio-oncology programs
2. What cardio-oncology services are currently offered at your institution?
   1. Consultation service operated by *general cardiologists* for assessment and management of cardio-toxicity among cancer patients
   2. Physicians specialized in the field of cardio-oncology who are comfortable managing cardio-toxic side effects due to cancer therapy and can give recommendations for adjusting cancer therapy
   3. Cardio-oncology services are not currently present and there *are no plans* to add these services
   4. Cardio-oncology services are not currently present but there *are* plans to add these services within the year
   5. I am unsure of the services that are available
3. In your opinion, what are the potential obstacles to the development of cardio-oncology units? You may select more than one option.
   1. Limited funding
   2. Limited infrastructure
   3. Limited need
   4. Limited interest
   5. No obstacle
   6. Other (please explain)

**SECTION IV – OPINIONS TOWARDS CURRENT PRACTICE**

1. In *your* opinion, on a scale of 1 to 5 (with 1 being very unimportant and 5 being very important), how important is it for *oncologists* to consider possible cardiac problems and cardio-toxic side effects when *planning* to initiate treatment for a cancer patient?
   1. Very unimportant
   2. Unimportant
   3. Neither important nor unimportant
   4. Important
   5. Very important
2. In *your* opinion, on a scale of 1 to 5 (with 1 being very unimportant and 5 being very important), how important is it for *oncologists* to consider possible cardiac problems and cardio-toxic side effects *during active cancer treatment*?
   1. Very unimportant
   2. Unimportant
   3. Neither important nor unimportant
   4. Important
   5. Very important
3. On a scale of 1 to 5 (with 1 being very unimportant and 5 being very important), how important is it for *oncologists* to consider possible cardiac problems due to cardiotoxicity in *cancer* *survivors* (patients with no active cancer who were treated 2 – 5 years ago)
   1. Very unimportant
   2. Unimportant
   3. Neither important nor unimportant
   4. Important
   5. Very important
4. In a patient with no underlying cardiac issues who is being started on a cancer therapy with potential cardiotoxic side effects, to what extent do you feel a cardiologist should be involved in their care?
   1. There is no need for involvement from a cardiologist during course of treatment
   2. Standard of care should include an assessment by a cardiologist
   3. Cardiology should provide *ongoing* monitoring for cardiotoxicity, even if the patient has no clinical symptoms of cardiac issues
   4. A cardiologist should be involved only when this patient develops active signs and symptoms of cardiotoxicity
5. On a scale of 1 to 5 (with 1 being strongly disagree and 5 being strongly agree), how strongly do you *personally* agree or disagree with this statement: “Cardioprotective medications should be standard of care for patients who are receiving cardiotoxic cancer therapy.
   1. Strongly disagree
   2. Disagree
   3. Neither agree nor disagree
   4. Agree
   5. Strongly agree
6. In your opinion, to what extent would access to a cardio-oncology service improve the prognosis of cancer patients?
   1. Access to a cardio-oncology service will significantly improve prognosis for cancer patients
   2. Access to a cardio-oncology service will not change prognosis for cancer patients
   3. Access to a cardio-oncology service will worsen prognosis for cancer patients
   4. I am unsure whether access to a cardio-oncology service will change prognosis

**SECTION V: CARDIOLOGISTS - UNDERSTANDING YOUR CURRENT PRACTICE**

Please answer the following questions **if you are a cardiologist**; if you are an **oncologist move on to SECTION VI**

1. On a scale of 1 to 5 (with 1 being strongly disagree and 5 being strongly agree), how strongly do you *personally* agree or disagree with this statement: I am knowledgeable about cardiovascular complications of cancer therapy.
   1. Strongly disagree
   2. Disagree
   3. Neither agree nor disagree
   4. Agree
   5. Strongly agree
2. On a scale of 1 to 5 (with 1 being strongly disagree and 5 being strongly agree), how strongly do you *personally* agree or disagree with this statement: I am comfortable treating cardiovascular complications of cancer therapy.
   1. Strongly disagree
   2. Disagree
   3. Neither agree nor disagree
   4. Agree
   5. Strongly agree
3. On a scale of 1 to 5 (with 1 being strongly disagree and 5 being strongly agree), how strongly do you *personally* agree or disagree with this statement: Oncologists are *knowledgeable* about cardiovascular complications of cancer therapy
   1. Strongly disagree
   2. Disagree
   3. Neither agree nor disagree
   4. Agree
   5. Strongly agree
4. On a scale of 1 to 5 (with 1 being strongly disagree and 5 being strongly agree), how strongly do you *personally* agree or disagree with this statement: Oncologists are *comfortable* with the management of cardiovascular complications of cancer therapy.
   1. Strongly disagree
   2. Disagree
   3. Neither agree nor disagree
   4. Agree
   5. Strongly agree
5. In your practice, on a scale of 1 to 5 (with 1 being never to 5 being always), how often do you prescribe *cardiac medications* to a patient with cardiotoxicity as a result of cancer therapy?
   1. Never
   2. Rarely
   3. Sometimes
   4. Very often
   5. Always

**SECTION VI: ONCOLOGISTS – UNDERSTANDING YOUR CURRENT PRACTICE**

Please answer the following questions **if you are an oncologist**. If you are a cardiologist, please proceed to **SECTION VII – CASES**

1. On a scale of 1 to 5 (with 1 being strongly disagree and 5 being strongly agree), how strongly do you *personally* agree or disagree with this statement: I am confident identifying cardiovascular complications of cancer therapy in my patients.
   1. Strongly disagree
   2. Disagree
   3. Neither agree nor disagree
   4. Agree
   5. Strongly agree
2. On a scale of 1 to 5 (with 1 being strongly disagree and 5 being strongly agree), how strongly do you *personally* agree or disagree with this statement: I feel *comfortable* treating cardiovascular complications of cancer therapy in my patients.
   1. Strongly disagree
   2. Disagree
   3. Neither agree nor disagree
   4. Agree
   5. Strongly agree
3. On a scale of 1 to 5 (with 1 being strongly disagree and 5 being strongly agree), how strongly do you agree or disagree with this statement: I have the *knowledge* to care for cardiovascular complications of cancer therapy in my patients.
   1. Strongly disagree
   2. Disagree
   3. Neither agree nor disagree
   4. Agree
   5. Strongly agree
4. On a scale of 1 to 5 (with 1 being strongly disagree and 5 being strongly agree), how strongly do you *personally* agree or disagree with this statement: Cardiologists are knowledgeable about cardiovascular complications of cancer therapy
   1. Strongly disagree
   2. Disagree
   3. Neither agree nor disagree
   4. Agree
   5. Strongly agree
5. On a scale of 1 to 5 (with 1 being strongly disagree and 5 being strongly agree), how strongly do you *personally* agree or disagree with this statement: Cardiologists are comfortable with the management of cardiovascular complications of cancer therapy.
   1. Strongly disagree
   2. Disagree
   3. Neither agree nor disagree
   4. Agree
   5. Strongly agree
6. As an oncologist, please estimate the proportion of patients for whom you would ask about cardiac disease and baseline cardiovascular risk factors *prior* to starting cancer therapy.
   1. 0%
   2. 1-25%
   3. 25-50%
   4. 50-75%
   5. 100%
7. In your practice, on a scale of 1 to 5 (with 1 being never to 5 being always), how often do you prescribe *cardiac medications* to a patient with cardiotoxicity as a result of cancer therapy?
   1. Never
   2. Rarely
   3. Sometimes
   4. Very often
   5. Always
8. Which resources do you primarily use for management of cardiotoxic side effects due to cancer therapies? Please select all that apply.
9. Data from clinical trials conducted for each cancer therapy
10. International guidelines published by expert societies
11. Information obtained at meetings by professional societies (e.g. CCS, ACS)
12. Colleagues specialized in cardio-oncology
13. Other (please specify)
14. Please estimate the percentage of patients whom you feel have not received optimal cancer treatment because of previous cardiovascular disease or risk factors for developing cardiotoxicity.
    1. <1% per year
    2. 1-5% per year
    3. 5-10% per year
    4. 10-20% per year
    5. >20% per year
15. At which EF would you interrupt ongoing cancer therapy for a patient who is receiving cancer therapy with *curative intent?*
    1. When the EF becomes <55%
    2. When the EF becomes 40-55%
    3. When the EF is < 40%
    4. A drop of >15% in EF
    5. If other, please specify
16. At which EF would you interrupt ongoing cancer therapy for a patient with *metastatic disease* who is receiving cancer therapy for palliation?
    1. When the EF becomes <55%
    2. When the EF becomes 40-55%
    3. When the EF is < 40%
    4. A drop of >15% in EF
    5. If other, please specify
17. In which clinical scenario(s) would you consider initiating *cardiac* treatment? Please select all that apply.
    1. Simultaneously with the *initiation* of a potentially cardiotoxic cancer treatment in a patient with normal EF at baseline (primary prevention)
    2. Simultaneously with the *initiation* of a potentially cardiotoxic cancer treatment if patient has an abnormal EF at baseline (prophylactic treatment)
    3. Abnormal or worsening of EF during cancer treatment
    4. Abnormal EF value *after* cancer treatment discontinuation or completion
18. How do you monitor for cardiotoxicity? Please select all that apply.
    1. Clinically, with focused history and physical examination
    2. With cardiac imaging at regular intervals
    3. With cardiac imaging ordered only when patients have symptoms concerning for cardiotoxicity
    4. By referral to cardiology for concurrent care during cancer therapy
    5. Other (please specify)
    6. I do not monitor for cardiotoxicity

**SECTION VII – CASES**

1. A 50year old female has received 12 cycles of trastuzumab/pertuzumab therapy for Her-2/neu positive metastatic breast cancer. Her ejection fraction at baseline was 55%, but on repeat echocardiogram decreased to 30%. She has no cardiac symptoms. What would be your management of the patient at this time?
   1. Continue trastuzumab/pertuzumab therapy at full dose
   2. Discontinue trastuzumab/pertuzumab therapy and refer to cardiology for further assessment
   3. Discontinue trastuzumab/pertuzumab therapy, repeat ECHO in a few weeks, and continue therapy at full dose if EF > 50 %.
   4. Discontinue trastuzumab therapy permanently
   5. If other, please specify
   6. I am unsure
2. A 58 year old male is receiving adjuvant infusional 5-fluorouracil during cycle 2 for resected stage III colorectal carcinoma. He develops sudden chest pain and nausea, and presents to the emergency department. A 12 lead electrocardiogram reveals inferior ST segment elevation. He is managed medically with complete resolution of symptoms. A subsequent angiogram reveals no evidence of coronary artery disease. A follow-up echocardiogram reveals an EF of 58%. What would you now recommend for adjuvant chemotherapy?
   1. Resume 5-fluorouracil at full dose
   2. Resume 5-fluorouracil at full dose but administer with cardiac monitoring
   3. Resume 5-fluorouracil at a reduced dose
   4. Hold 5-FU and refer to cardiology
   5. Change chemotherapy to oral capecitabine
   6. Change chemotherapy to intravenous raltitrexed
   7. Discontinue adjuvant chemotherapy
   8. I am unsure
   9. If other, please specify
